# Commissioning of an expanded-field radiation technique using a gimbal-mounted linear accelerator

**DOI:** 10.1016/j.phro.2026.101013

**Published:** 2026-06-03

**Authors:** Tomohiro Ono, Fumiya Tanaka, Shunta Jinno, Tetsuo Fukuda, Hiroyuki Kato, Hideaki Hirashima, Yuka Ono, Mitsuhiro Nakamura, Takashi Mizowaki

**Affiliations:** a Department of Radiation Oncology and Image-Applied Therapy, Graduate School of Medicine, Kyoto University, Kyoto, Japan; b Department of Radiation Oncology, Shiga General Hospital, Shiga, Japan; c X-Ray Therapy Division, Therapy System Business, Healthcare Business Group, Hitachi HighTech Corporation, Tokyo, Japan; d Department of Advanced Medical Physics, Graduate School of Medicine, Kyoto University, Kyoto, Japan

**Keywords:** OXRAY, Gimbal-mounted linear accelerator, Treatment planning system, Expanded-field, Radiation technique, Commissioning, Quality assurance

## Abstract

**Background and purpose:**

A novel gimbal-mounted linear accelerator (linac) enables off-axis beam delivery. This study was aimed at commissioning an expanded-field radiation technique using a gimbal-mounted linac to validate beam data and implement patient-specific quality assurance (QA).

**Materials and methods:**

Measurements were performed with gimbal head rotations of ±50.0 mm at the isocenter plane in both pan and tilt directions and were compared with the treatment planning system (TPS) calculation results. Percentage depth doses (PDDs) were obtained for multi-leaf collimator-shaped field sizes. Off-center ratios (OCRs) were measured at the depth of maximum dose (16 mm for 6 MV and 6 MV-FFF beams) and at depths of 50, 100, and 200 mm. For patient-specific QA, film-based measurements were conducted in patients with breast cancer involving axillary lymph nodes and esophageal cancer.

**Results:**

For the PDDs and OCRs, differences between the measured and TPS-calculated doses were maintained within ±2% inside the flattening region at all energy levels under gimbal head rotation. For patient-specific QA, gamma passing rates at 3%/2 mm exceeded 90% for each case.

**Conclusions:**

These findings demonstrate that the proposed technique enables precise and effective off-axis beam delivery, thereby advancing the capabilities of modern radiotherapy.

## Highlights

• A novel expanded-field radiation technique was successfully commissioned.
• Beam-positioning accuracy with gimbal head rotation was 0.47 ± 0.21 mm.
• Percentage depth doses and off-center ratios were maintained within ±2%.
• Gamma passing rates at 3%/2 mm exceeded 90% in all cases.
• The proposed technique enables precise and effective off-axis beam delivery.

## Graphical abstract

**Figure:**
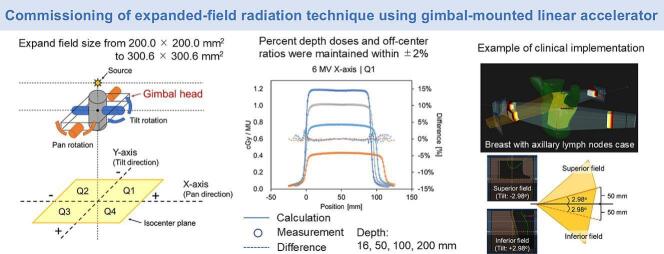

## 1 Introduction

Radiation therapy is a cornerstone modality in cancer treatment, and its technological advancements have been notable. High-precision techniques such as intensity-modulated radiation therapy (IMRT) and image-guided radiation therapy are widely incorporated into modern treatment systems, significantly improving local control rates while reducing adverse events across various malignancies [1], [2].

Gimbal-mounted linear accelerators enable real-time tumor tracking and highly conformal dose delivery via continuously non-coplanar irradiation [3], [4], [5]. However, such systems are constrained by their maximum field dimensions (typically 150 mm × 150 mm to 200 mm × 200 mm) owing to mechanical limitations, which limit their applicability in treating larger target volumes and extensive treatment areas. For example, real-time tumor tracking has been reported for relatively small targets, such as the lung, liver, and pancreas [6], [7], [8]. Similarly, continuous non-coplanar irradiation has been applied to limited treatment sites, including partial breast irradiation, localized lung cancer, prostate, and bone metastases, reflecting a bias in treatment sites due to restrictions in irradiation field size [9], [10]. Therefore, expansion of the irradiation field is necessary to broaden applicability to a wider range of treatment sites.

This limitation was addressed by developing an expanded-field technique using gimbal head rotation [11], [12], which extends the radiation field to 300.6 mm × 300.6 mm (original field: 200 mm × 200 mm), potentially enabling treatment of larger sites such as head and neck, breast with axillary nodes, and esophagus. Previous studies have demonstrated the hardware-level feasibility of these techniques; however, gimbal head rotation was reproduced using a traditional general-purpose treatment planning system (TPS), which did not inherently support gimbal head rotation. A key limitation was the lack of a TPS capable of accounting for this rotation [11], [12]. In recent years, gimbal planning functionality has been implemented in a commercial TPS, allowing treatment planning that incorporates gimbal head rotation [13], thereby enabling TPS-based evaluation that accurately reflects gimbal head rotation.

This study investigated the expanded-field radiation technique for a new gimbal-mounted linac to validate beam data and conduct patient-specific quality assurance (QA) for expanded-field delivery.

## 2 Materials and methods

### 2.1 Features of the gimbal head

**Fig. 1:**
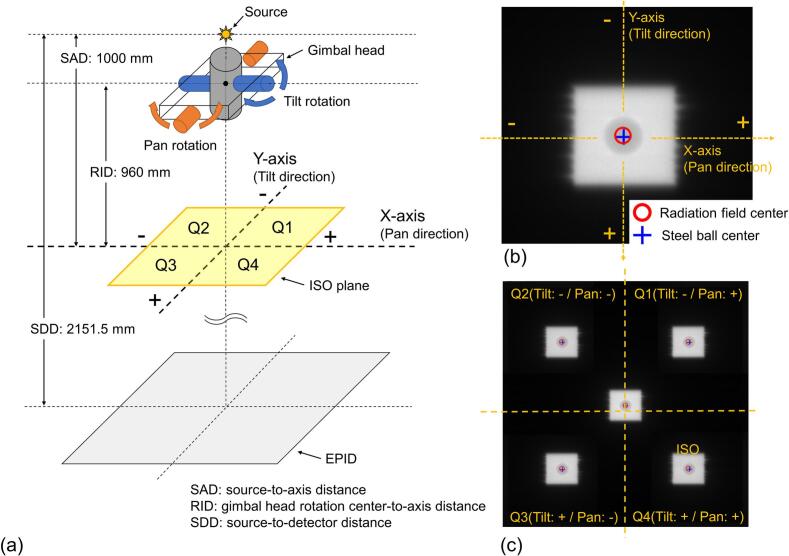
Simplified geometric model of the gimbal-mounted linear accelerator (a). Schematic illustration of the Winston-Lutz test on an EPID image (b). Circles and crosses indicate the centers of the radiation field and steel ball, respectively. Representative EPID images for five irradiation conditions, including the ISO, Q1, Q2, Q3, and Q4 (c).

Investigations were performed using OXRAY system (Hitachi Ltd., Tokyo, Japan) as a gimbal-mounted linear accelerator. The OXRAY system had a source-to-axis distance (SAD) of 1000 mm and a gimbal head rotation center-to-axis distance of 960 mm (Fig. 1 (a)). The available beam energies were 6 MV and 6 MV-FFF. The treatment aperture was a multi-leaf collimator (MLC), with leaf widths of 2.5 mm in the central 40-mm region around the isocenter (ISO) and 5 mm in the remaining region. The nominal maximum MLC field size was 200 mm × 200 mm. The gimbal head rotated along two orthogonal axes, pan and tilt, up to ±3.00°, thereby enabling the beam to swing up to ±50.3 mm from the ISO in each direction within the plane perpendicular to the beam and achieve a maximum MLC field size of 300.6 mm × 300.6 mm.

The expanded-field radiation technique was simulated in RayStation for research purposes (version 2023B; RaySearch Laboratories, Stockholm, Sweden). The TPS allows gimbal angles or corresponding displacement inputs in the pan and tilt directions at the ISO plane. Dose calculation does not require beam data acquired with gimbal head rotation, because the gimbal head structure remains unchanged, and the beam direction vector is modified through coordinate transformation based on pan- and tilt-axis rotations (Fig. 1(a)). Off-axis characteristics were not extrapolated from isocenter commissioning data. Because the physical characteristics of the beam are invariant, no additional beam data is required for gimbal-based planning. Here, dose calculations were performed using the collapsed cone algorithm with a grid of dimensions 2.0 mm × 2.0 mm × 2.0 mm.

### 2.2 Geometric validation of the beam-positioning accuracy

The beam-positioning accuracy of the expanded-field radiation technique was evaluated using the Winston–Lutz test [14] and in-house software developed in MATLAB 2023b (MathWorks Inc., Natick, MA, USA). A water-equivalent cube phantom (60 mm × 60 mm × 60 mm) containing a centrally embedded 10-mm-diameter steel ball was placed on the treatment couch. An MLC-shaped square field of 30 mm × 30 mm was generated using the OXRAY system with 6 MV energy.

Beam-positioning accuracy was defined as the difference between the centroid of the radiation field and the center of the steel ball, as detected by the electronic portal imaging device (EPID), shown in Fig. 1(a). The circles and crosses indicate the centers of the radiation field and steel ball, respectively. The EPID of the OXRAY system was set at a source-to-detector distance of 2151.5 mm and had a pixel pitch of 0.278 mm on the imager plane (Fig. 1(a)). Therefore, the EPID had a pixel pitch of 0.129 mm at the ISO level and an image resolution of 1536 × 1536 pixels with 2 × 2 binning. Beam-positioning accuracy was subsequently evaluated at gantry angles of 0°, 90°, 180°, and 270°. Accuracy was also evaluated with gimbal head rotations of ±2.98° (corresponding to ±50.0 mm at the ISO plane) in both the pan and tilt directions at each gantry angle. Sag correction was automatically applied by the OXRAY system.

In the EPID image plane, quadrants were defined based on pan and tilt signs: Q1 (+pan, –tilt), Q2 (–pan, –tilt), Q3 (–pan, +tilt), and Q4 (+pan, +tilt) (Fig. 1). These positions were collectively defined as off-ISO positions. The gimbal head rotation center was aligned with the cube phantom by shifting the treatment couch ±50.0 mm in the direction of gimbal head rotation. Supplementary Tables S1 and S2 provide concise summaries of the geometric offsets in the pan and tilt directions, respectively. EPID images were acquired at five positions, one ISO and four off-ISO positions, across the four gantry angles, with five repeated measurements per setup, resulting in a total of 100 EPID images. The mean and standard deviation (SD) of the beam-positioning accuracy were subsequently calculated.

### 2.3 Dosimetric validation of beam data

The phantom was positioned with a source-to-surface distance (SSD) of 900 mm and a SAD of 1,000 mm. Percentage depth doses (PDDs) were measured for MLC-shaped fields of dimensions 50 mm × 50 mm, 100 mm × 100 mm, and 200 mm × 200 mm. Off-center ratios (OCRs) were measured at the depth of maximum dose (16 mm) and at depths of 50, 100, and 200 mm.

All measurements were performed using 6 MV and 6 MV-FFF beams from the OXRAY system, with gimbal head rotations of ±50.0 mm at the ISO plane in both the pan and tilt directions. Data were acquired using a SmartScan system (IBA Dosimetry, Schwarzenbruck, Germany) and an ionization chamber (CC04; IBA Dosimetry), which is suitable for beam scanning as recommended by IAEA TRS 430 [15] and AAPM Medical Physics Practical Guideline (MPPG) 5.a. [16]. Scanning was performed at a continuous scanning speed of 3.0 mm/s, with the detector oriented perpendicular to the beam central axis. The normalization point was the depth of maximum dose (16 mm) for PDD measurements and the central axis for OCR measurements.

For off-axis measurements, the phantom remained at the ISO, and measurements were performed by moving the detector to the center of the off-axis position. Measurements were obtained at the ISO and in all four quadrants. The measured data were evaluated according to the European Society for Radiotherapy and Oncology (ESTRO) Booklet 7 [17]. For PDDs, the evaluation metrics included the high-dose/low-dose gradient area at the central beam axis (*δ*_1_) and the build-up region of the central beam axis (*δ*_2_). For OCRs, the evaluated parameters included the penumbra region (*δ*_2_), outer central beam axis region with a high-dose/low-dose gradient (*δ*_3_), outer beam edges with a low-dose/low-dose gradient (*δ*_4_), radiological width with a high-dose/high-dose gradient (RW_50_), and beam fringe with a high-dose/high-dose gradient (*δ*_50–90_).

### 2.4 Dosimetric validation of the experimental measurements using films

**Fig. 2:**
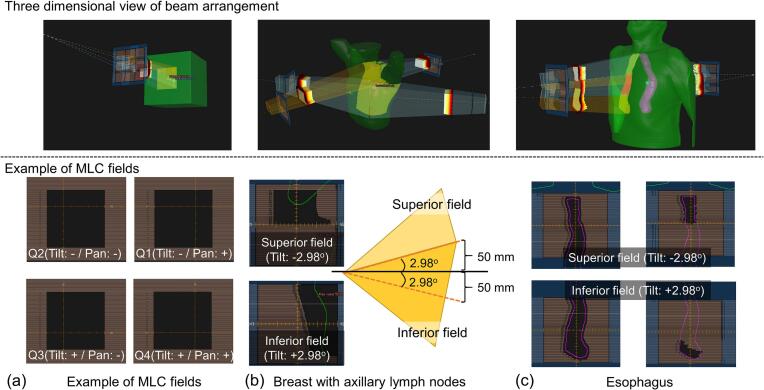
Beam arrangements for the rectangular field created by combining four piecewise fields (a), breast irradiation including axillary lymph nodes generated using superior and inferior fields (b), and esophageal irradiation generated using superior and inferior fields with the field-in-field technique (c). The upper panels show three-dimensional views of the beam arrangements, whereas the lower panels present examples of the corresponding MLC fields.

The expanded field was generated by combining multiple fields using gimbal head rotation and evaluated using EBT4 radiochromic film (Ashland Inc., Wayne, NJ, USA). EBT4 has been reported to exhibit a variation of 10 cGy at 200 cGy [18]. In this study, 14 dose points ranging from 0 to 1370.3 cGy were used to generate dose–response curves, and a linear fitting model was applied. Film scanning was performed using an Epson DS-G30000 scanner in transmission mode, with 48-bit uncompressed red, green, and blue tagged image file format images (16 bits per color channel) at a resolution of 75 dots per inch in professional mode. The film was scanned in landscape orientation. Three irradiation conditions were assessed: a rectangular field and two clinical cases involving breast cancer with axillary lymph nodes and esophageal cancer (Fig. 2). Dose distributions were assessed using the gamma passing rate (GPR) with a 3%/2 mm criterion, calculated using dosimetric software (DD system, ver. 17.252; R'Tech Inc., Tokyo, Japan). The passing rate was determined for regions within the 10% isodose level using global normalization. A rectangular field was created by combining four piecewise fields [11], [12], each comprising an asymmetric 50 mm × 50 mm MLC-shaped field, with an inner field width of 50 mm (Fig. 2(a)). The field junctions were optimized to achieve a uniform dose profile at the ISO plane. The gimbal head was rotated by ±2.98° in the tilt direction (equivalent to ±50.0 mm at the ISO plane) and by ±3.00° in the pan direction (equivalent to ±50.3 mm at the ISO plane), accounting for MLC round-leaf transmission. The evaluated field dimensions were 100 mm × 100 mm, determined based on the dimensions of the EBT4 film. The phantom was positioned with an SSD of 900 mm and an SAD of 1000 mm. EBT4 films were placed at depths of 50, 100, and 150 mm in a water-equivalent slab phantom (TM Phantom; Taisei Medical Co. Ltd., Osaka, Japan).

For patient-specific QA, two clinical cases involving breast cancer with axillary lymph nodes and esophageal cancer were investigated. For the breast cancer case, a partially wide tangential field was generated using gimbal head rotation in the tilt direction (Fig. 2(b)). The junction region was aligned on the ISO plane in the same manner as that used for the rectangular field. Superior fields were designed as half fields with a head rotation of −2.98°, whereas inferior fields were arranged as tangential fields with a head rotation of +2.98° in the tilt direction. Fig. 2(b) shows a schematic illustrating the geometric relationship between the two fields.

For the esophageal cancer case, a field-in-field technique was created using the automatic field-in-field planning function implemented in RayStation, with gimbal head rotations of ±2.98° in the tilt direction (Fig. 2(c)). Dose distribution was evaluated using EBT4 films inserted into the sagittal and coronal planes of an IMRT phantom (IBA Dosimetry, Schwarzenbruck, Germany) to assess the junction regions.

## 3 Results

**Fig. 3:**
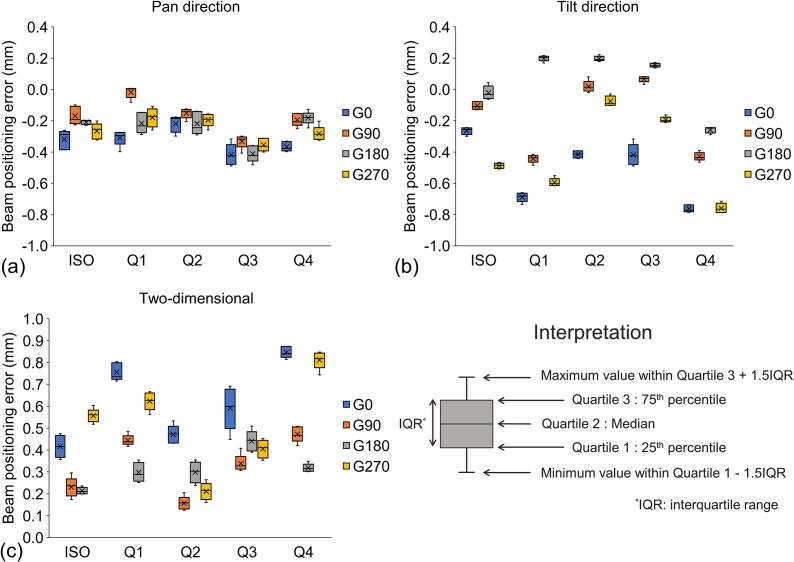
Boxplots of beam-positioning errors in the pan direction (a), tilt direction (b), and two-dimensional evaluation (c).

Beam-positioning accuracy was 0.35 ± 0.15 mm at the ISO and 0.47 ± 0.21 mm at 50 mm off-ISO positions under Q1 to Q4 (Fig. 3). Beam-positioning accuracies were consistently larger at off-ISO positions than at the ISO.

**Fig. 4:**
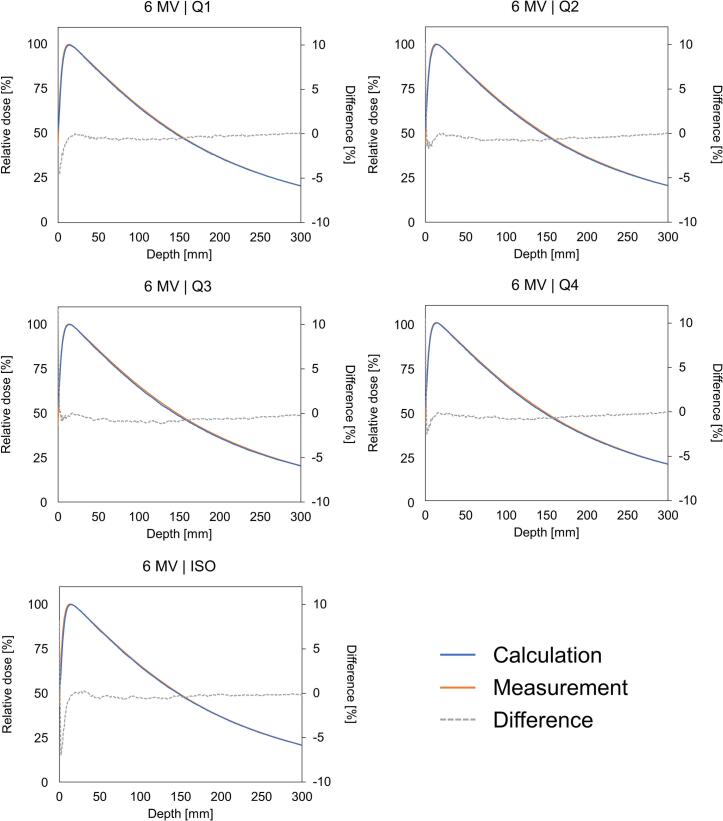
Percentage depth doses for 6 MV beams under each expanded-field irradiation condition. The MLC-shaped field had dimensions of 100 mm × 100 mm. Calculated and measured doses, along with their differences, are shown.

**Fig. 5:**
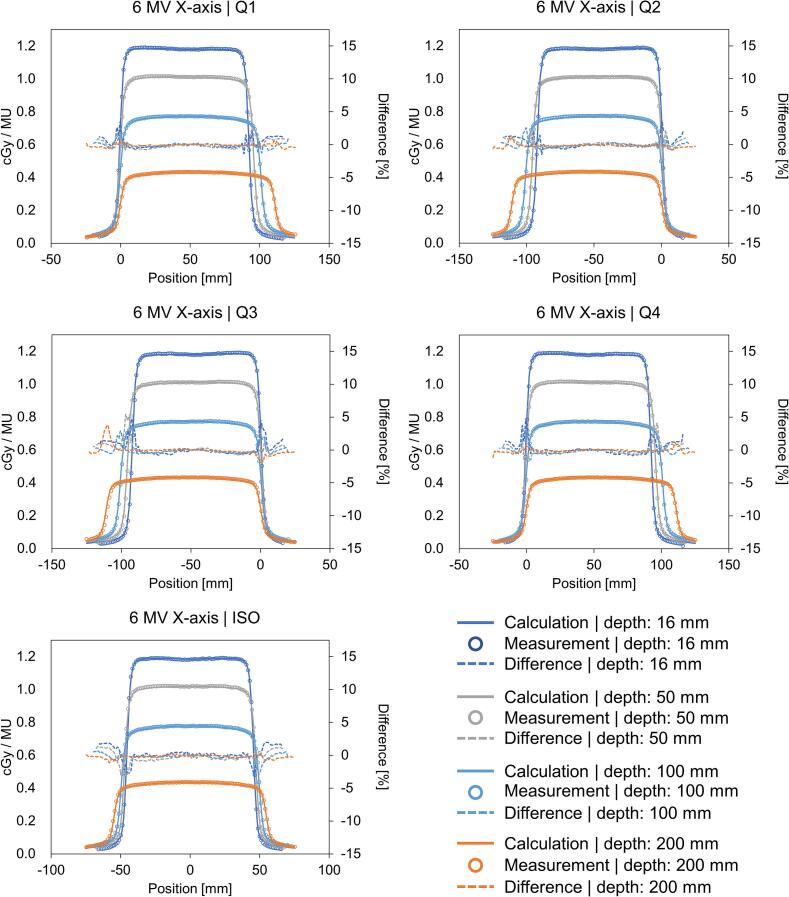
Off-center ratios for 6 MV beams at various depths under each expanded-field irradiation condition along the X-axis. The MLC-shaped field had dimensions of 100 mm × 100 mm. Calculated and measured doses, along with their differences, are shown.

For 6-MV beams, the PDDs showed minimal variation between ISO and Q1–Q4 conditions for MLC-shaped fields of dimensions ranging from 50 mm × 50 mm to 200 mm × 200 mm (Fig. 4, and Supplementary Fig. S1, S2 and S3). The maximum values of *δ*_1_ and *δ*_2_ are within tolerance level (Supplementary Tables S3 and S4). Similar trends were observed for 6 MV-FFF beams. OCRs along the X-axis demonstrated that beam profiles under Q1–Q4 conditions were comparable to those at the ISO, despite the oblique incidence introduced by gimbal head rotation (Fig. 5, and Supplementary Figs. S4, S5 and S6). The maximum values of *δ*_2_, *δ*_3_, *δ*_4_, RW_50_, and *δ*_50–90_ are also within tolerance level (Supplementary Tables S5 and S6).

**Fig. 6:**
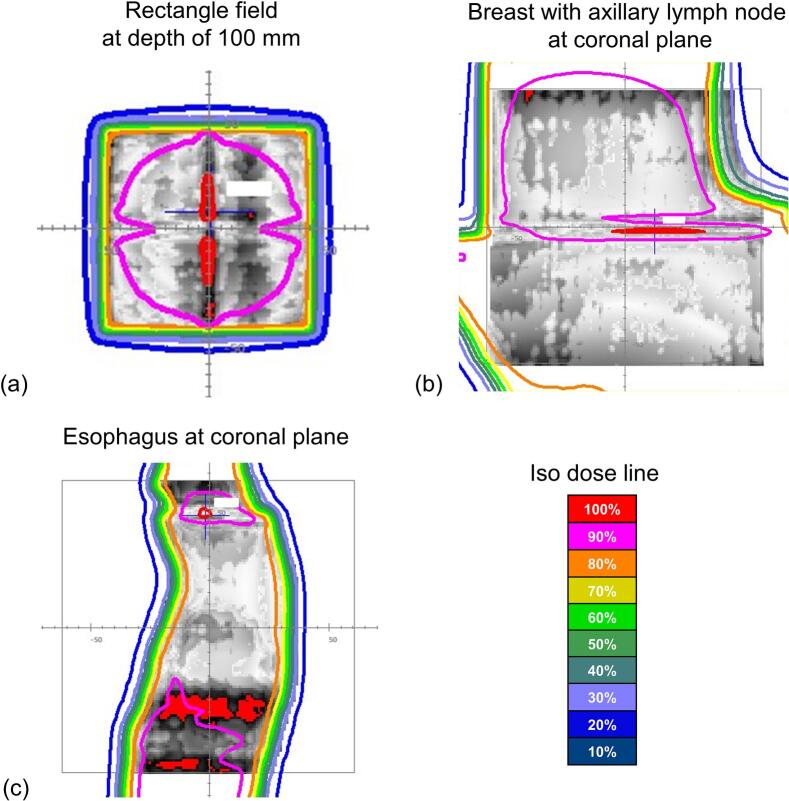
Dose distributions of gamma passing rate for a rectangular field (a), breast cancer involving axillary lymph nodes (b), and esophageal cancer (c).

For the rectangular field, dose differences within the junction region were maintained within ±5% in both the tilt and pan directions (Supplementary Fig. 7). The corresponding GPRs at 3%/2 mm ranged from 91.7% to 96.3% (Fig. 6). In the breast cancer case, the junction region was located approximately 4 mm inferior to the center, resulting in GPRs of 95.8% and 93.2% in the sagittal and coronal planes, respectively. For the esophageal cancer case, the junction region was located approximately 40 mm superior to the center. Across both clinical cases, dose differences within the junction regions remained within ± 5%, while GPRs at 3%/2 mm ranged from 93.2% to 97.9%. All evaluated cases achieved GPRs exceeding 90% at the 3%/2 mm criterion.

## 4 Discussion

This study reported the commissioning of the expanded-field radiation technique. The results demonstrated high beam-positioning accuracy, sufficient beam model accuracy, and deliverable patient-specific QA outcomes. These findings support the feasibility of the proposed approach and its potential contribution to the clinical application of OXRAY technology.

Beam-positioning accuracy was evaluated to verify the safety of high-precision radiotherapy. The gimbal mechanism operates in two modes: stationary mode, with a fixed gimbal angle, and dynamic mode, with continuous variation of the gimbal angle. These modes exhibit different beam-positioning accuracy characteristics. In this study, the stationary mode was evaluated for expanded-field irradiation. The mean ± standard deviation (SD) of the beam-positioning accuracy was 0.35 ± 0.15 mm at the ISO and 0.47 ± 0.21 mm at positions 50 mm off-ISO under the expanded-field irradiation condition. For comparison, Ono et al. reported Winston-Lutz test results at off-isocenter (off-IC) positions using the TrueBeam STx (Varian Medical Systems) [19], with beam-positioning errors of 0.50 ± 0.23 mm (maximum: 1.1 mm) at positions 50 mm from the ISO in quadrants Q1-Q4. These findings indicate that the gimbal-mounted OXRAY system achieves positioning accuracy comparable to that of a conventional C-arm-type linac. Similarly, Kido et al. evaluated beam-positioning accuracy at off-ISO positions for commercial C-arm linacs using a custom phantom across multiple institutions [20]. They reported beam-positioning errors ranging from 0.62 to 1.19 mm at distances up to 70 mm off-ISO. Therefore, the gimbal-mounted linac demonstrated superior off-ISO positioning accuracy compared with conventional C-arm linacs.

PDDs showed no significant differences between conditions with and without gimbal-driven beam delivery, as the calculated and measured values demonstrated close agreement despite the oblique geometry. In contrast, the OCRs obtained using the expanded-field radiation technique exhibited beam profile characteristics typical of oblique irradiation. For oblique beams, comparison of dose profiles or selected reference points in the calculated distribution has been recommended [21], [22]. In this study, the agreement between the calculated and measured PDDs and OCRs confirmed the accuracy of dose calculations for the expanded-field radiation technique. This successful validation demonstrated that RayStation accurately reproduced the expanded-field radiation technique, supporting its integration into routine clinical practice.

When implementing expanded-field irradiation with a commercial C-arm linac, the treatment couch is typically repositioned using a multiple-ISO technique [23], [24]. However, couch movement introduces setup errors that may cause dose inconsistencies at the junction regions between treatment fields, representing a major clinical concern. For setup errors of ±3 mm in multiple-ISO techniques, studies by Seppälä et al. [25] and Hadley et al. [26] reported dose variations at junction fields of approximately ±10% to 15%. Ono et al. investigated junction regions using the expanded-field radiation technique with the Vero4DRT system [11] and reported dose deviations ranging from −0.9% to 5.0% in a water-equivalent phantom at depths of 50, 100, and 150 mm. Consistent with these findings, the present study observed no substantial discrepancies at the junction regions, with deviations between calculated and measured values remaining within ±5%. Compared with conventional multiple-ISO techniques, the expanded-field radiation technique using a gimbal-mounted linac relies on the positional accuracy of the gimbal and MLC for field junction alignment. This approach eliminates patient-related setup errors and therefore offers a substantial clinical advantage.

In the future, treatment planning using the expanded-field radiation technique is expected to be extended to other radiation modalities, such as volumetric modulated arc therapy (VMAT). Additionally, the gimbal-mounted linac enables biaxially rotational dynamic radiation therapy (BROAD-RT), which combines simultaneous gantry rotation with the O-ring structure [9], [10], [27], [28], [29], [30], [31]. BROAD-RT has been reported to improve dose distribution, including planning target volume homogeneity and conformity, while reducing doses to organs at risk compared with coplanar VMAT in nasopharyngeal carcinoma [13]. Because the expanded-field radiation technique and BROAD-RT operate through independent control mechanisms, their integration represents a promising approach for advancing next-generation radiation therapy.

This study has a limitation in that commissioning of the expanded-field radiation technique was performed under fixed irradiation conditions. Two primary approaches to field expansion using a gimbal have been proposed: one based on multiple static segments and the other involving continuous gimbal rotation. However, no commercial TPS currently supports simulation of dose distributions during continuous rotation for the expanded-field radiation technique. If irradiation techniques incorporating dynamic gimbal rotation become clinically available, several potential advantages may be achieved. These approaches are currently under investigation and may further expand the clinical capabilities of gimbal-mounted linac systems [12]. Addressing this limitation could facilitate the development of a more efficient field-expansion method.

In summary, the expanded-field radiation technique was successfully commissioned on a gimbal-mounted linac, demonstrating high accuracy in beam data validation and patient-specific QA. Differences between the measured and calculated values remained within clinically acceptable limits, confirming the reliability of the system. These findings highlight the potential of this technique to enable precise and effective off-axis beam delivery, thereby advancing the capabilities of modern radiotherapy.

## CRediT authorship contribution statement

**Tomohiro Ono:** Writing – original draft, Validation, Software, Methodology, Conceptualization. **Fumiya Tanaka:** Writing – review & editing, Validation, Methodology, Investigation. **Shunta Jinno:** Writing – review & editing, Validation, Methodology, Investigation. **Tetsuo Fukuda:** Writing – review & editing, Validation, Methodology, Investigation. **Hiroyuki Kato:** Writing – review & editing. **Hideaki Hirashima:** Writing – review & editing. **Yuka Ono:** Writing – review & editing. **Mitsuhiro Nakamura:** Writing – review & editing, Supervision, Project administration, Conceptualization. **Takashi Mizowaki:** Writing – review & editing, Supervision, Project administration, Conceptualization.

## Funding

This study was supported by the 10.13039/100009619Japan Agency for Medical Research and Development (JP24ck0106924) and collaborative research agreements with Hitachi High-tech Corporation.

## Declaration of competing interest

The authors declare the following financial interests/personal relationships which may be considered as potential competing interests: This study was funded by Hitachi High-tech. Takashi Mizowaki and Mitsuhiro Nakamura report receiving collaborative research contracts and scholarship donations, as well as a speaker fee, from Hitachi High-Tech. The other co-authors are involved in collaborative research with Hitachi High-Tech.

## References

[1] Bujold A., Craig T., Jaffray D., Dawson L.A. (2012). Image-guided radiotherapy: has it influenced patient outcomes?. Semin Radiat Oncol.

[2] Chandra R.A., Keane F.K., Voncken F.E.M., Thomas C.R. (2021). Contemporary radiotherapy: present and future. Lancet.

[3] Kamino Y., Takayama K., Kokubo M., Narita Y., Hirai E., Kawawda N. (2006). Development of a four-dimensional image-guided radiotherapy system with a gimbaled X-ray head. Int J Radiat Oncol Biol Phys.

[4] Hiraoka M., Mizowaki T., Matsuo Y., Nakamura M., Verellen D. (2020). The gimbaled-head radiotherapy system: rise and downfall of a dedicated system for dynamic tumor tracking with real-time monitoring and dynamic WaveArc. Radiother Oncol.

[5] Kawata K., Kishigami Y., Hirashima H., Sawada Y., Urago M., Fujimoto T. (2025). Performance evaluation of a second-generation O-ring-shaped image-guided radiotherapy system with a gimbal-mounted linear accelerator and real-time tracking capabilities. J Appl Clin Med Phys.

[6] Matsuo Y., Hiraoka M., Karasawa K., Kokubo M., Sakamoto T., Mukumoto N. (2022). Multi-institutional phase II study on the safety and efficacy of dynamic tumor tracking-stereotactic body radiotherapy for lung tumors. Radiother Oncol.

[7] Iizuka Y., Inoue M., Kokubo M., Sakamoto T., Murofushi K.N., Imagumbai T. (2025). Long-term results of dynamic tumor-tracking stereotactic body radiotherapy with real-time monitoring using a gimbal-mounted linac for liver tumors: a multicenter observational study. Int J Clin Oncol.

[8] Yoshimura M., Hiraoka M., Kokubo M., Sakamoto T., Karasawa K., Matsuo Y. (2025). Multi-institutional phase II study on the efficacy and safety of dynamic tumor-tracking, moderately hypofractionated intensity-modulated radiotherapy in patients with locally advanced pancreatic cancer. Cancer Med.

[9] Burghelea M., Verellen D., Dhont J., Hung C., Gevaert T., Van den Begin R. (2017). Treating patients with dynamic wave arc: first clinical experience. Radiother Oncol.

[10] Ono Y., Yoshimura M., Hirata K., Ono T., Hirashima H., Mukumoto N. (2018). Dosimetric advantages afforded by a new irradiation technique, dynamic WaveArc, used for accelerated partial breast irradiation. Phys Med.

[11] Ono T., Miyabe Y., Yamada M., Yokota K., Kaneko S., Sawada A. (2014). Development of an expanded-field irradiation technique using a gimbaled X-ray head. Med Phys.

[12] Ono T., Miyabe Y., Yokota K., Takahashi K., Akimoto M., Mukumoto N. (2016). Development of a gimbal-swing irradiation technique for uniform expanded-field, wedged-beam, and intensity-modulated radiation therapy. Biomed Phys Eng Express.

[13] Hiraoka S., Hirashima H., Nakamura M., Tanaka F., Adachi H., Ono Y. (2025). Integration test of biaxially rotational dynamic-radiation therapy for nasopharyngeal carcinoma: efficacy evaluation and dosimetric analysis. Radiother Oncol.

[14] Lutz W., Winston K.R., Maleki N. (1988). A system for stereotactic radiosurgery with a linear accelerator. Int J Radiat Oncol Biol Phys.

[15] International Atomic Energy Agency. Commissioning and quality assurance of computerized planning systems for radiation treatment of cancer. IAEA Technical Reports Series No. 430. Vienna: IAEA; 2004.

[16] Smilowitz J.B., Das I.J., Feygelman V., Fraass B.A., Kry S.F., Marshall I.R. (2015). AAPM Medical Physics Practice Guideline 5.a.: commissioning and QA of treatment planning dose calculations – megavoltage photon and electron beams. J Appl Clin Med Phys.

[17] ESTRO Booklet No. 7. Quality assurance of treatment planning systems: Practical examples for non-IMRT photon beams. Brussels: ESTRO; 2004.

[18] Miura H., Ozawa S., Okazue T., Enosaki T., Nagata Y. (2023). Characterization of scanning orientation and lateral response artifact for EBT4 Gafchromic film. J Appl Clin Med Phys.

[19] Ono T., Kido T., Nakamura M., Iramina H., Kakino R., Mizowaki T. (2023). Automatic measurement of beam-positioning accuracy at off-isocenter positions. J Appl Clin Med Phys.

[20] Kido T., Ono T., Nakamura M., Ishihara Y., Itoh H., Matsugi K. (2023). Development and multi-institutional evaluation of a new phantom for verifying beam-positioning errors at off-isocenter positions. Phys Med.

[21] Sharpe M.B. (2006). IAEA Technical Reports Series No. 430: commissioning and quality assurance of computerized planning systems for radiation treatment of cancer. Med Phys.

[22] Jacqmin D.J., Bredfeldt J.S., Frigo S.P., Smilowitz J.B. (2017). Implementation of the validation testing in MPPG 5.a “commissioning and QA of treatment planning dose calculations-megavoltage photon and electron beams”. J Appl Clin Med Phys.

[23] Lee Y.K., Brooks C.J., Bedford J.L., Warrington A.P., Saran F.H. (2012). Development and evaluation of multiple isocentric volumetric modulated arc therapy technique for craniospinal axis radiotherapy planning. Int J Radiat Oncol Biol Phys.

[24] Zhang Y., Huang Y., Lin J., Ding S., Gong X., Liu Q. (2023). Multi-isocenter VMAT craniospinal irradiation using feasibility dose-volume histogram-guided auto-planning technique. J Radiat Res.

[25] Seppala J., Kulmala J., Lindholm P., Minn H. (2010). A method to improve target dose homogeneity of craniospinal irradiation using dynamic split field IMRT. Radiother Oncol.

[26] Hadley A., Ding G.X. (2014). A single-gradient junction technique to replace multiple-junction shifts for craniospinal irradiation treatment. Med Dosim.

[27] Mizowaki T., Takayama K., Nagano K., Miyabe Y., Matsuo Y., Kaneko S. (2013). Feasibility evaluation of a new irradiation technique: three-dimensional unicursal irradiation with the Vero4DRT (MHI-TM2000). J Radiat Res.

[28] Burghelea M., Verellen D., Poels K., Hung C., Nakamura M., Dhont J. (2016). Initial characterization, dosimetric benchmark and performance validation of Dynamic Wave Arc. Radiat Oncol.

[29] Miura H., Nakao M., Doi Y., Ozawa S., Kenjo M., Nagata Y. (2022). Treatment planning comparison between dynamic wave arc and volumetric modulated arc therapies for prostate-cancer treatment. Med Dosim.

[30] Hirashima H., Adachi H., Ono T., Nakamura M., Ono Y., Iwai T. (2025). Determination of patient-specific trajectory for biaxially rotational dynamic-radiation therapy using a new O-ring-shaped image guided radiotherapy system. Phys Imaging Radiat Oncol.

[31] Hirotaki K., Tomizawa K., Kitou S., Jinno S., Moriya S., Fujisawa T. (2025). Dosimetric comparison of noncoplanar VMAT without rotating the patient couch versus conventional coplanar/noncoplanar VMAT for head and neck cancer: first report of Dynamic Swing Arc. Adv Radiat Oncol.

