# Supplementary material for: Commissioning of an expanded-field radiation technique using a gimbal-mounted linear accelerator
Source: Phys Imaging Radiat Oncol. 2026 Jun 3;39:101013. doi: 10.1016/j.phro.2026.101013 (PMC13264245; doi:10.1016/j.phro.2026.101013)
Supplement: Supplementary Data 1 [file mmc1.pdf]

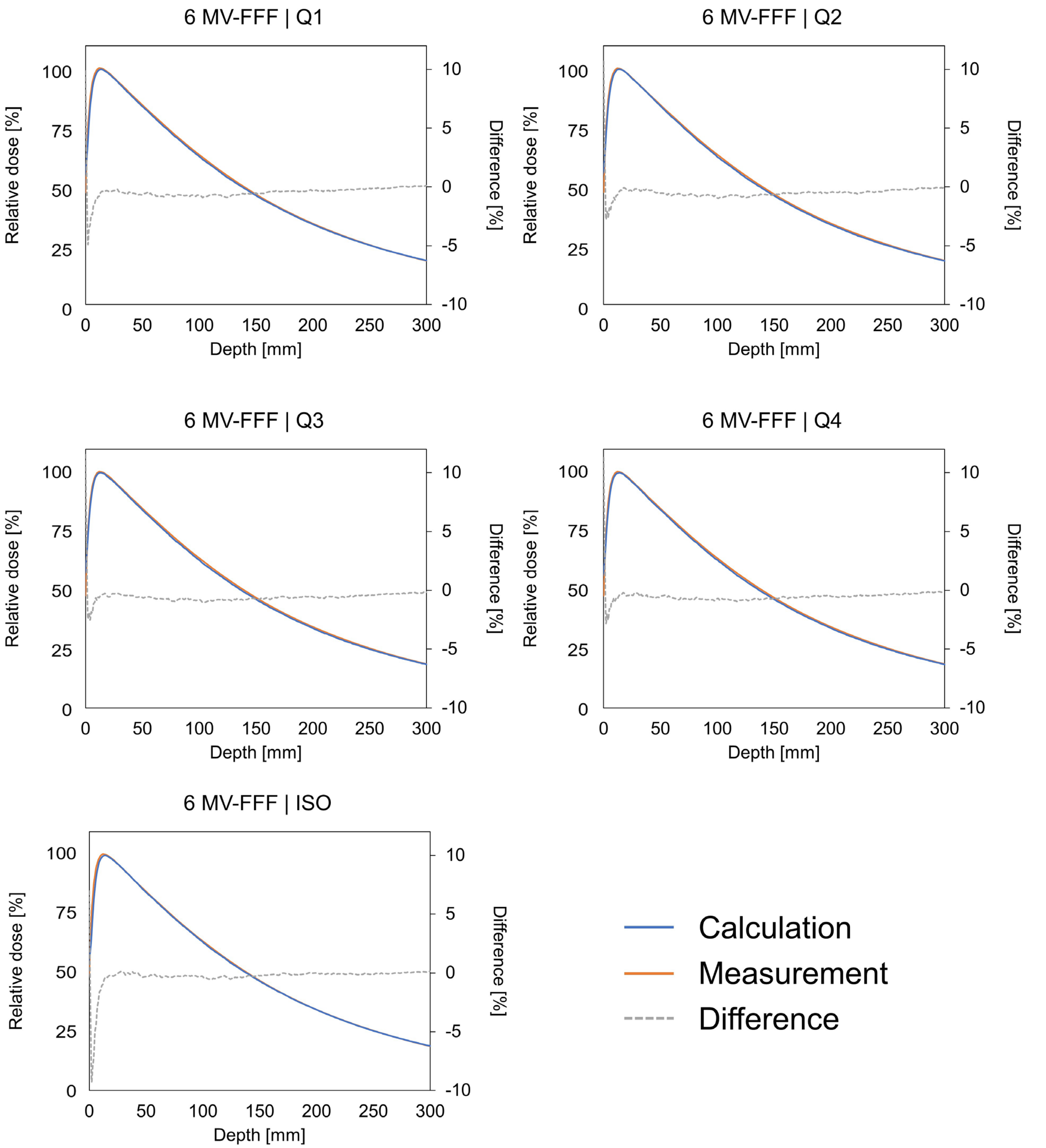

Supplementary Figure S1 Percentage depth doses of 6 MV-FFF beams using the expanded-field radiation technique for an MLC-shaped field size of 100 × 100 mm^2. The calculated and measured doses and their differences are shown.

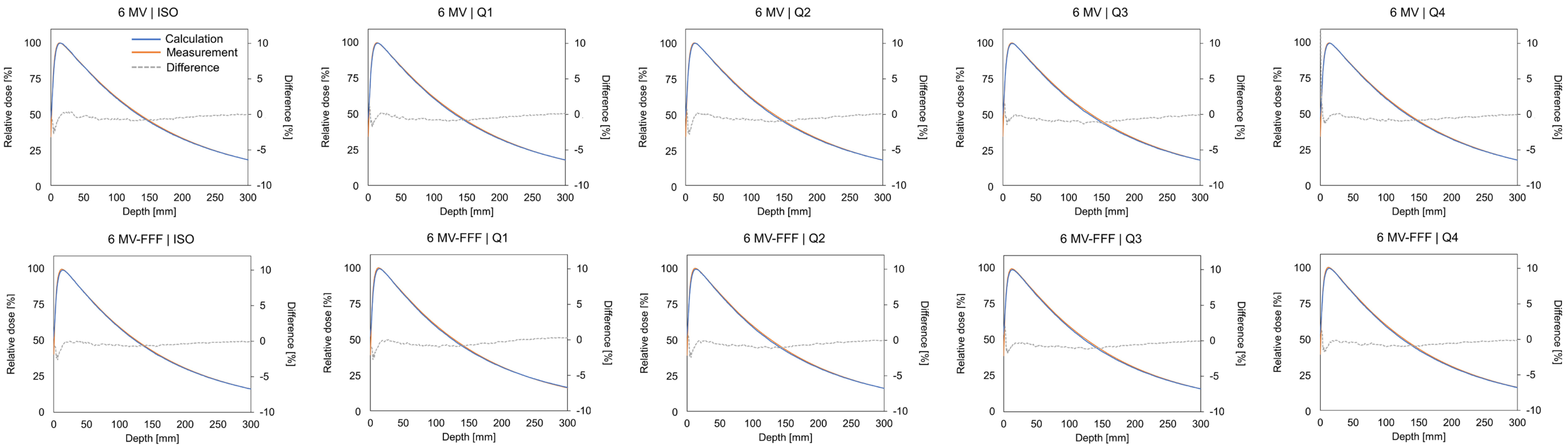

Supplementary Figure S2 Percentage depth doses of the 6 MV and 6MV-FFF beams using the expanded-field radiation technique. The MLC-shaped field size was 50 × 50 mm^2. The calculated and measured doses and their differences are shown.

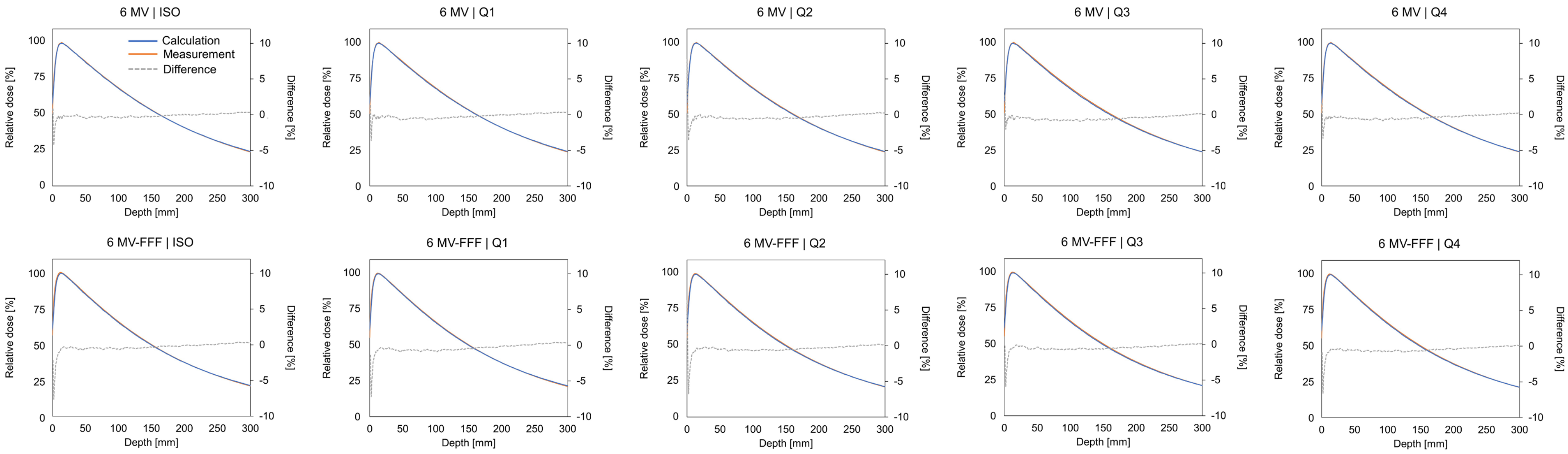

Supplementary Figure S3 Percentage depth doses of the 6 MV and 6 MV-FFF using the expanded-field radiation technique. The MLC-shaped field size was 200 × 200 mm^2. The calculated and measured dose and their differences are shown.

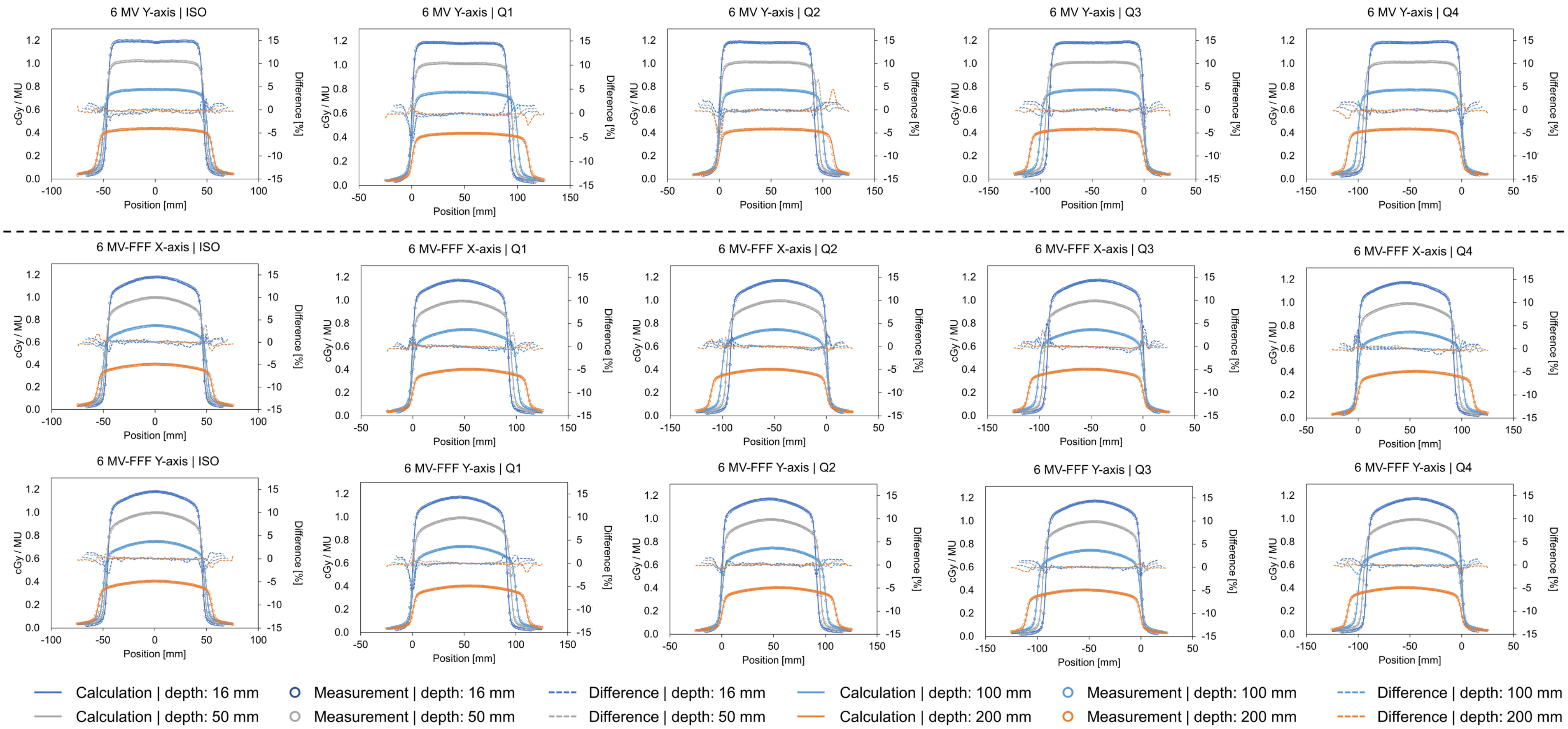

Supplementary Figure S4 Off-center ratios of the 6 MV and 6 MV-FFF beams at each depth using the expanded-field radiation technique. The MLC-shaped field size was 100 × 100 mm^2. The calculated and measured doses and the differences are shown.

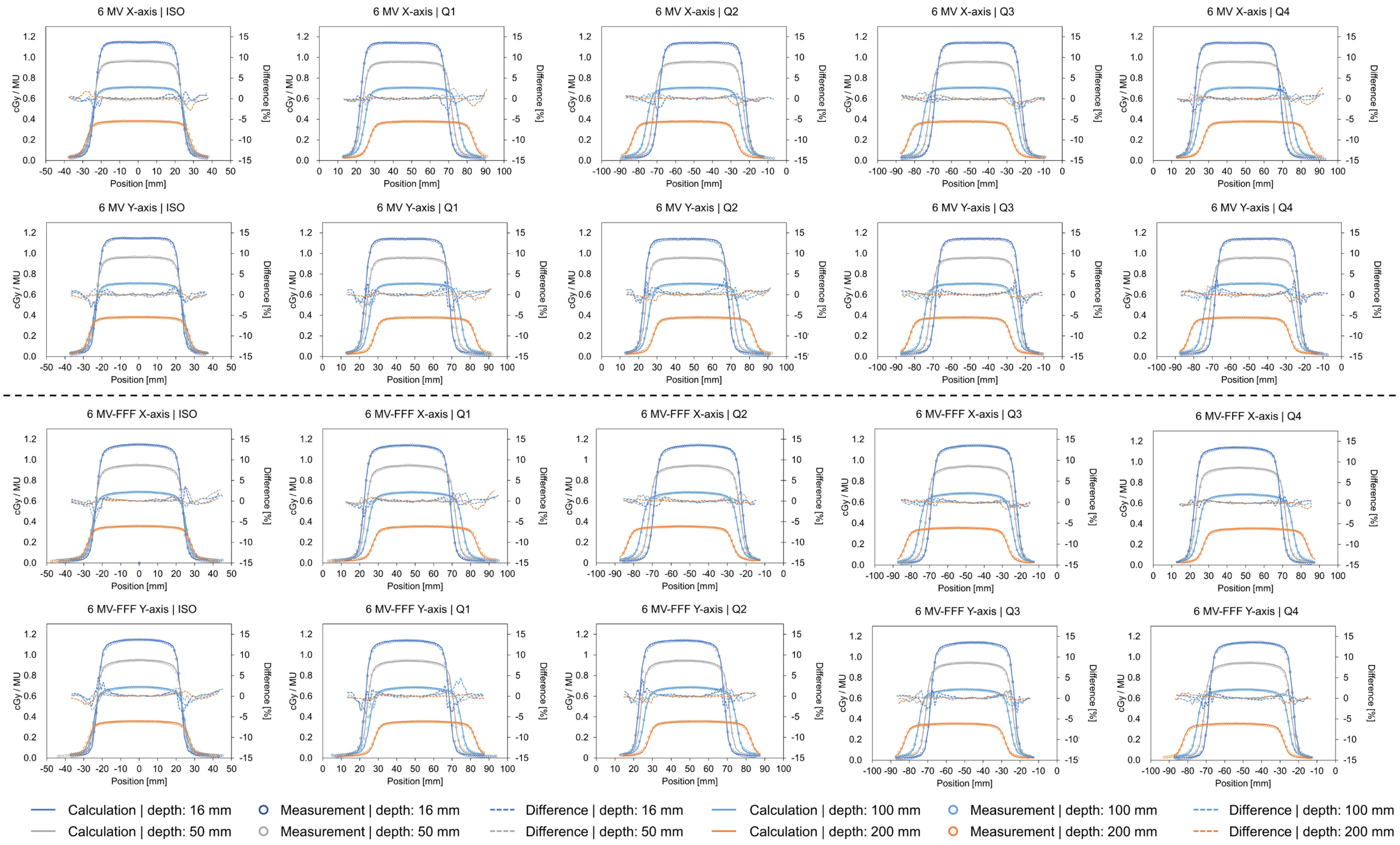

Supplementary Figure S5 Off-center ratios of the 6 MV and 6 MV-FFF beams at each depth using the expanded-field radiation technique. The MLC-shaped field size was 50 × 50 mm^2. The calculated and measured doses and the differences are shown.

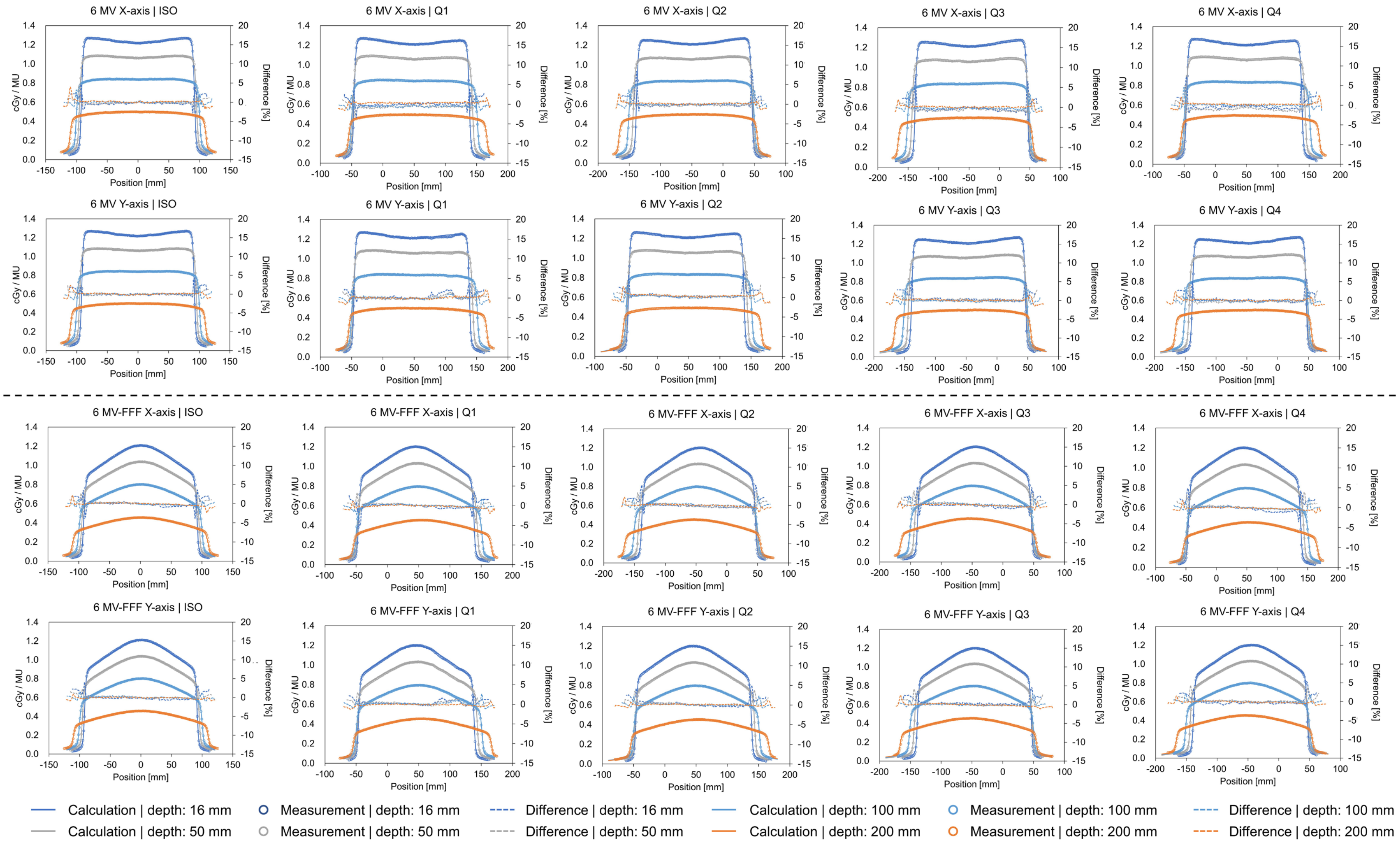

Supplementary Figure S6 Off-center ratios of the 6 MV and 6 MV-FFF beams at each depth using the expanded-field radiation technique. The MLC-shaped field size was 200 × 200 mm^2. The calculated and measured doses and the differences are shown.

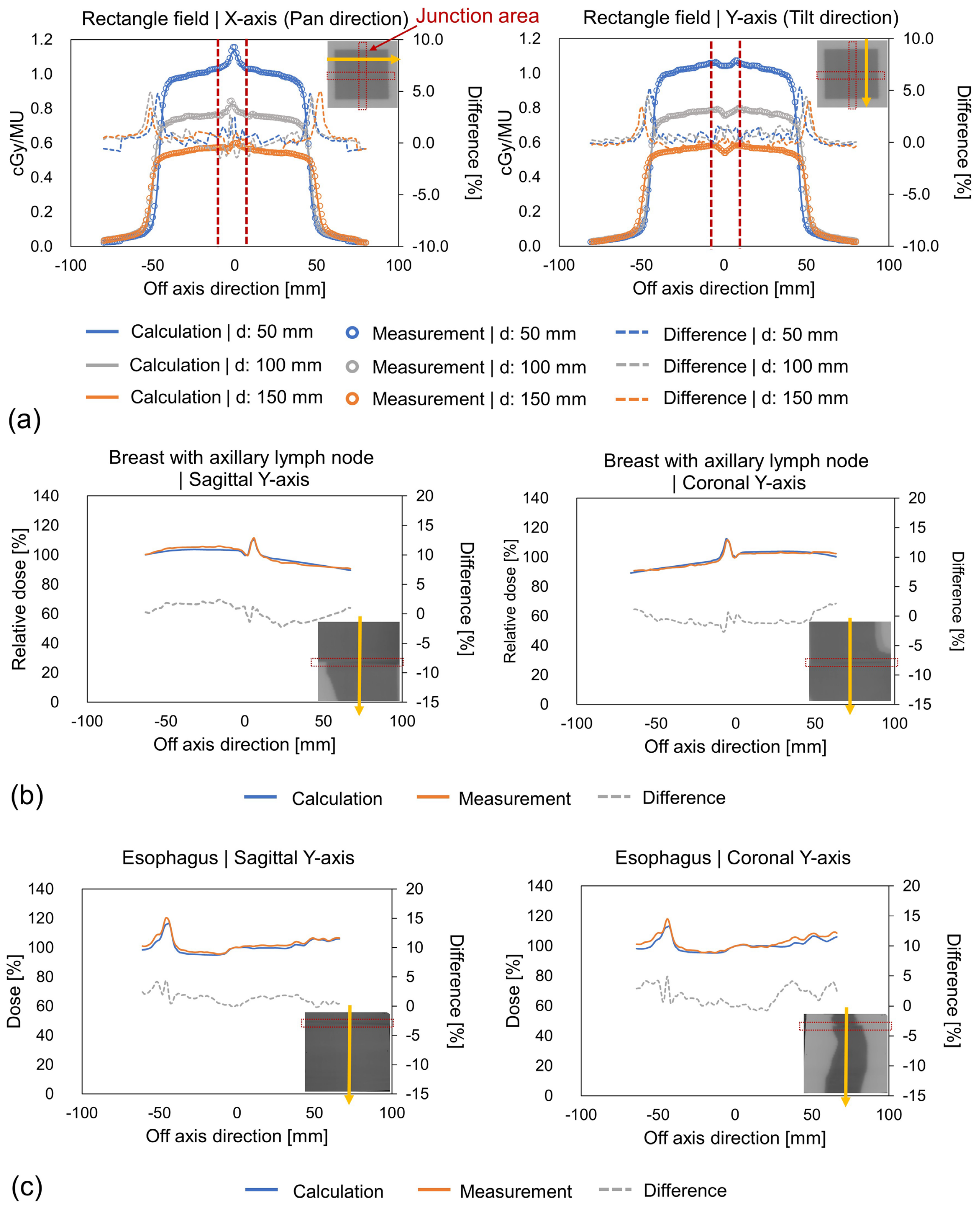

Supplementary Figure S7 Dose profiles from the experimental measurements of a rectangular field (a), breast with axillary lymph nodes (b), and esophageal cancer (c).

Supplementary Table S1 Geometric offset relationship between pan rotation and the X-axis.

	Pan [°]								
	-3.00	-2.98	-2.00	-1.00	0.00	1.00	2.00	2.98	3.00
X-axis [mm]	-50.3	-50.0	-33.5	-16.8	0.0	16.8	33.5	50.0	50.3

Supplementary Table S2 Geometric offset relationship between tilt rotation and the Y-axis.

	Tilt [°]								
	-3.00	-2.98	-2.00	-1.00	0.00	1.00	2.00	2.98	3.00
Y-axis [mm]	50.3	50.0	33.5	16.8	0.0	-16.8	-33.5	-50.0	-50.3

Supplementary Table S3 Summary of the maximum δ_1 and δ_2 values in PDD for 6 MV beams.

Energy	Field size [mm^2]		Tolerance level	ISO	Q1	Q2	Q3	Q4
6 MV	50 × 50	δ_1 [%]	2	1.0	0.9	1.1	1.3	1.0
		δ_2 [%]	10	1.6	1.8	2.0	1.4	1.3
		δ_2 [mm]	2	1.0	0.9	0.5	0.5	0.3
	100 × 100	δ_1 [%]	2	0.9	0.8	0.9	1.2	0.9
		δ_2 [%]	10	2.2	4.5	1.7	0.9	2.4
		δ_2 [mm]	2	0.4	0.6	0.3	0.5	0.4
	200 × 200	δ_1 [%]	2	0.6	0.8	0.7	0.9	0.9
		δ_2 [%]	10	2.7	3.6	3.5	1.9	3.2
		δ_2 [mm]	2	0.7	0.6	0.6	0.1	0.7

Supplementary Table S4 Summary of the maximum δ_1 and δ_2 values in PDD for 6 MV-FFF beams.

Energy	Field size [mm^2]		Tolerance level	ISO	Q1	Q2	Q3	D4
6 MV-FFF	50 × 50	δ_1 [%]	2	0.8	1.0	1.2	1.2	1.0
		δ_2 [%]	10	2.0	2.8	2.4	1.8	1.7
		δ_2 [mm]	2	0.7	0.5	0.6	0.8	0.7
	100 × 100	δ_1 [%]	2	0.9	0.9	1.0	1.0	0.9
		δ_2 [%]	10	1.6	4.8	2.7	2.5	2.7
		δ_2 [mm]	2	0.8	0.7	0.6	0.8	0.7
	200 × 200	δ_1 [%]	2	0.5	0.7	0.5	0.5	0.6
		δ_2 [%]	10	3.5	3.0	3.3	3.9	3.7
		δ_2 [mm]	2	0.5	0.5	0.5	0.6	0.6

Supplementary Table S5 Summary of the maximum δ_2, δ_3, δ_4, RW_{50}, and δ_{50-90} values in OCR for 6 MV beams.

Energy	Field size [mm^2]		Tolerance level	Crossline					Inline				
				ISO	Q1	Q2	Q3	Q4	ISO	Q1	Q2	Q3	Q4
6 MV	50 × 50	δ_2 [%]	10	8.4	5.9	9.2	8.7	8.2	7.9	9.7	8.6	9.1	9.4
		δ_2 [mm]	2	0.6	0.1	0.0	0.1	0.0	0.2	0.3	0.3	0.5	0.4
		δ_3 [%]	3	1.4	1.0	1.2	1.0	0.9	1.6	1.3	1.9	1.8	1.8
		δ_4 [%]	3	1.0	0.9	0.6	0.7	1.3	1.0	1.1	1.3	0.8	0.8
		RW_{50} [mm]	2	0.3	0.3	0.2	0.3	0.2	0.2	0.2	0.1	0.1	0.1
		$\delta_{50\text{-}90}$ [mm]	2	0.4	0.2	0.2	0.1	0.3	0.8	0.3	0.3	0.4	0.3
	100 × 100	δ_2 [%]	10	6.9	7.5	7.9	9.7	8.4	8.7	9.8	7.6	7.6	8.9
		δ_2 [mm]	2	0.3	0.4	0.8	1.0	0.6	0.4	0.5	0.3	0.4	0.2
		δ_3 [%]	3	0.6	1.1	0.9	0.8	0.9	1.1	1.9	1.1	0.9	1.7
		δ_4 [%]	3	0.6	0.7	0.6	0.7	0.7	0.7	0.9	0.8	0.6	0.6
		RW_{50} [mm]	2	0.2	0.2	0.8	0.5	0.4	0.3	0.4	0.1	0.5	0.3
		$\delta_{50\text{-}90}$ [mm]	2	0.3	0.4	0.4	0.3	0.3	0.1	0.7	0.2	0.2	0.6
	200 × 200	δ_2 [%]	10	9.7	8.6	9.9	9.9	9.9	9.3	9.5	9.8	9.8	9.6
		δ_2 [mm]	2	2.0	1.5	1.3	1.8	2.0	1.8	1.2	0.7	1.2	1.5
		δ_3 [%]	3	1.7	1.8	1.6	1.4	1.7	0.9	1.8	1.9	1.2	1.3
		δ_4 [%]	3	1.1	1.1	0.4	0.5	1.7	1.6	1.8	0.7	0.7	0.7
		RW_{50} [mm]	2	0.5	0.5	0.8	0.8	1.0	0.4	0.5	0.7	0.7	0.6
		$\delta_{50\text{-}90}$ [mm]	2	1.8	1.8	1.3	0.8	1.1	1.5	1.9	1.8	0.8	1.4

Supplementary Table S6 Summary of the maximum δ_2, δ_3, δ_4, RW_{50}, and $\delta_{50\text{–}90}$ values in OCR for 6 MV-FFF beams.

Energy	Field size [mm^2]		Tolerance level	Crossline					Inline				
				ISO	Q1	Q2	Q3	Q4	ISO	Q1	Q2	Q3	Q4
6 MV-FFF	50 × 50	δ_2 [%]	10	3.2	8.8	7.5	9.1	6.6	7.9	9.4	9.1	8.9	4.6
		δ_2 [mm]	2	0.5	0.3	0.6	0.2	0.1	1.0	0.3	1.3	0.5	0.4
		δ_3 [%]	3	1.2	1.6	1.3	1.0	1.3	1.6	1.5	1.8	1.6	2.0
		δ_4 [%]	3	0.6	1.7	1.2	0.6	0.9	0.9	0.8	0.9	0.6	0.7
		RW_{50} [mm]	2	0.2	0.4	0.5	0.2	0.1	0.3	0.2	0.1	0.1	0.0
		$\delta_{50\text{-}90}$ [mm]	2	0.5	0.2	0.3	0.3	0.2	0.6	0.5	0.3	0.3	0.7
	100 × 100	δ_2 [%]	10	9.7	8.7	9.8	9.4	9.7	9.5	8.4	6.8	8.2	8.7
		δ_2 [mm]	2	0.5	0.4	0.4	0.2	1.0	0.3	0.4	0.8	0.9	0.7
		δ_3 [%]	3	1.3	1.0	1.0	1.1	1.4	0.9	0.9	0.8	0.9	1.1
		δ_4 [%]	3	0.8	0.5	0.5	0.6	0.7	0.6	0.8	0.6	0.6	0.5
		RW_{50} [mm]	2	0.0	0.2	0.3	0.8	0.4	0.4	0.5	0.2	0.1	0.3
		$\delta_{50\text{-}90}$ [mm]	2	0.3	0.8	1.0	0.6	0.5	0.5	0.6	0.2	0.3	0.3
	200 × 200	δ_2 [%]	10	8.2	7.7	9.6	6.7	8.5	7.6	8.2	9.0	8.8	9.3
		δ_2 [mm]	2	1.7	0.9	1.7	1.1	0.8	1.5	1.8	1.8	1.3	1.9
		δ_3 [%]	3	1.7	0.8	1.1	1.0	1.2	1.5	1.6	1.2	1.6	0.8
		δ_4 [%]	3	0.8	1.0	1.1	1.1	1.1	0.6	1.1	0.8	1.0	1.6
		RW_{50} [mm]	2	0.4	0.6	0.3	0.2	0.3	0.4	0.5	0.9	0.8	0.6
		$\delta_{50\text{-}90}$ [mm]	2	1.0	1.5	1.3	0.5	1.7	1.4	2.0	0.5	0.7	1.4
